# Group Treatment for Women Gamblers Using Web, Teleconference and Workbook: Effectiveness Pilot

**DOI:** 10.1007/s11469-016-9700-4

**Published:** 2016-09-08

**Authors:** Roberta R. Boughton, Farah Jindani, Nigel E. Turner

**Affiliations:** 1 Problem Gambling Institute of Ontario, Centre for Addiction and Mental Health, Toronto, ON Canada; 2 Education Services, Centre for Addiction and Mental Health, Toronto, ON Canada; 3 Centre for Addiction and Mental Health, Institute for Mental Health Policy Research, 33 Russell Street, Toronto, ON M5S 2S1 Canada; 4 Dalla Lana School of Public Health, University of Toronto, Toronto, ON Canada

**Keywords:** Women and gambling, Problem gambling treatment, Self-help work book, Teleconference, Remote treatment

## Abstract

While the past decades have seen a dramatic increase in the number of women who gamble and develop consequent problems, treatment services are being underutilized in Ontario. This pilot study explores the feasibility of using web- and phone-based group interventions to expand services available for women who might not otherwise seek or be able to access treatment. Distinct treatment considerations for working with women, such as the value of a women’s group, advantages of phone counselling, and the implementation of modern web-based services, were reviewed. The study involved a clinician-facilitated group that used teleconferencing and webinar technology (Adobe Connect) for support and discussion, and a Tutorial Workbook (TW) developed specifically to address the issues and treatment needs of women who gamble at a problematic level. A mixed method analysis used to evaluate the results suggested that the group-based teleconference/webinar approach provided a much-needed means of treatment support for women. Participants reported that the program helped them to understand their gambling triggers, to improve their awareness, to feel better about themselves, to modify their mood and anxiety levels, to feel less isolated, to address their relationships, and to feel more hopeful for the future. The Tutorial Workbook, which was used to supplement the educational component of the group interaction, was highly rated.

Previous research indicates that women face a number of barriers to on-site outpatient treatment for problem gambling, including many practical concerns such as time constraints (work and caretaking demands), need for childcare, financial limitations, and travel obstacles Boughton and Brewster (2002); Gainsbury et al. 2014; Suurvali et al. 2009). Especially applicable in small communities, barriers also include psychological issues such as social stigma and a fear of recognition, judgment or exposure. Mental health issues such as depression and anxiety prevent women some from seeking help. And key among the findings of Boughton and Brewster (2002) was considerable ambivalence, a fear that treatment would demand total abstinence, and a belief on the part of the women gamblers that they should be able to make changes on their own.

Suurvali et al. (2008) argue the value of providing a range of treatment options as a means to circumvent obstacles to treatment, noting the “growing body of literature supporting the effectiveness of brief interventions for gambling problems offered by telephone or the internet or in the form of self-help workbooks” (p. 1345). This statement captures the focus of the pilot study reported in this paper.

This treatment intervention follows on the findings of an earlier study by Boughton and Brewster (2002). Our study was a pragmatic clinical trial (Tse et al. 2013) offering resources and support to women who might not otherwise seek or be able to access treatment. Women took part in a clinician-facilitated group treatment using teleconferencing in conjunction with webinar technology (Adobe Connect) and a Tutorial Workbook designed by the first author.

The rationale as to the choice of treatment intervention necessitates a review of our current knowledge about the need for treatment tailored to women’s special needs and issues, the value of a women’s group, the increasingly utilized role and advantages of phone counselling, and the modern implementation of web based services. Boughton and Brewster (2002) explored women’s service needs, confirming the diversity of approaches helpful to women who gamble, which included phone counselling and a strong preference for women-only over mixed group supports. Research suggests that treatment is commonly tailored to the needs and interests of male gamblers (Crisp et al. 2000; Mark and Lesieur 1992). Treatment needs and appropriate approaches for women are, however, different (Albanese et al. 2011; Grant and Kim 2002; Ibáñez et al. 2003; Thomas and Moore 2003; Toneatto and Wang 2009). In particular, female gamblers need to have their specific affective symptoms or emotional needs attended to (Grant and Kim 2002; Ibáñez et al. 2003; Potenza et al. 2001).

Mark and Lesieur (1992) suggested that mixed gender groups can be less effective for some women because of a “masculine tilt” (p. 556). As Deborah Smith, executive director of the California Women’s Commission on Alcoholism (cited by Underhill 1986, p.47).states: “In mixed groups, men talk about their problems. The women support the men. The men get better, the women don’t”. Co-ed treatment can impede successful outcomes because of women’s common histories of harmful or painful relationships with men (Underhill 1986; Wilke 1994). Women’s groups offer freedom and increased comfort to talk about personal and/or women specific issues such as intimacy, pregnancy or violence. Women learn to value themselves and other women. They understand and share similar experiences. Evidence shows that a women-only treatment group “produces positive results for women in terms of increased self-esteem and sense of personal power” (Wilke 1994, p. 32). Piquette-Tomei et al. (2007) write of women only groups as havens of safety and support, reporting qualitative research findings that such groups were perceived to be central to the effectiveness of working through the myriad issues related to problem gambling. This process of normalizing, sharing and supporting one another are critical therapeutic factors in change (Yalom 1985).

## Phone Access to Counselling

Another finding from Boughton and Brewster (2002) was that women endorsed telephone counselling as a helpful treatment option, recommending flexible hours of service, including evening hours and weekends. The experiences of Helplines confirm the importance of phone support in reducing treatment barriers; telephone contact is almost always the first mode of intervention for those seeking help (Tse et al. 2013). Available 24/7, Problem Gambling Helplines provide supportive counselling and information about treatments, providing a conduit for linking potential consumers to resources. Rodda and Lubman (2014) report that 70 % of people accessing Gambling Help Online Service in Australia were seeking treatment for the first time. Over 70 % of people accessing real time chat did so during evening, overnight or weekend periods.

Limited research exists, however, into the effectiveness of phone counselling as an independent treatment option for gamblers, and no studies to date examine the value of phone counselling specific to women gamblers. However, a recent study by Tse et al. (2013) compared the effectiveness of face-to-face and telephone counselling for problem gambling with a predominantly female sample (67 %) that involved six telephone or face-to-face interventions over a 3 month period. Researchers claim preliminary evidence that both forms of counselling are effective: No significant differences were found between the pre and post intervention measures in comparing the groups.

Other treatment methods involving phone counselling individually and in groups (Telehealth, Telecoaching) have been studied in the field of mental health research (Brenes et al. 2011; Coman et al. 2001; Evans et al. 1984; IZAAK 2013; Reese et al. 2002). Benson (2008) offers a 12 week Telecoaching group intervention for shopping addiction. While its effectiveness has yet to be compared to face to face group counselling (personal communication Jan. 2015), Benson promotes the coaching group as offering a combination of peer support, encouragement and feedback under the guidance of an expert. The Free Yourself Program, developed by Gabriela Byrne (1999), addresses problem gambling. This program, based on neuro-linguistic programming (a method of influencing brain behaviour through the use of language to enable a person to “recode” the way the brain responds to stimuli) offers both phone counselling and group support sessions (seminars).

There are advantages and disadvantages to phone counselling. Easy access is the most cited advantage (Tse et al. 2013). It saves time and travel costs, affords flexibility of participation with minimal disruption and offers immediacy of contact. Anonymity is another advantage, often having special appeal to those clients experiencing shame and fear of identification. Phone counseling seems to help people express their views and feelings more easily, and equalize the power relationship (Brenes et al. 2011; Coman et al. 2001). In addition to observing that telephone counselling is cost effective, Brenes et al. (2011) report both high levels of client satisfaction and lower rates of attrition in studies comparing telephone to face-to face counselling.

The challenges to conducting psychotherapy by telephone include issues of confidentiality and privacy, ethics and legality in some parts of the world (Brenes et al. 2011). The management of crisis situations such as suicidal behaviours or worsening symptoms are considerations. Brenes et al. (2011) writes, however, that the largest issue is psychotherapists’ openness to working in a non-traditional manner. The resistance stems from the perception that psychotherapy is more difficult because of the lack of nonauditory cues, distraction, the risk of the session becoming more social than therapeutic and concerns about therapeutic alliance (see also Tse et al. 2013).

Phone counselling requires a highly developed clinical ear for communications including an awareness of broadcast through tone, speed of speech, timbre, intensity, breathing, silence and language (Coman et al. 2001). At the same time, based on the clinical experience of the first author, advantages to phone counselling exist. The absence of visual distractions enables focused attention solely on the subtleties of the content and presentation: what is said, what is not said, and how it is said. Moreover the more superficial social aspects of the clinician’s (or clients’) comportment cease to distract.

Phone counselling may not be optimal for all clients, for example, if motivation is low, such as with mandated clients (Brenes et al. 2011). Reese et al. (2002) note concern about the its effectiveness for clients who began counselling feeling very poorly, reporting that these clients showed less improvement. (It is unknown, however, if these same clients would have achieved more improvement in a face to face encounter). The counselling agency used in their study typically referred callers with more severe or chronic mental health issues to local mental health services so as to avoid becoming a crisis line. To address this concern, Reese et al. (2002) recommend assessing the client’s level of functioning to determine suitability for telephone treatment. On site crisis management (such as taking the client to the emergency department if decompensating) may be an advantage of face to face counselling. With phone counselling Brenes et al. (2011) stress developing a safety plan at the onset, and having a safety protocol in place that is understood by the client. This includes a clear explanation of the limits to confidentiality and an informed consent to treatment.

It is argued that phone interventions have a valuable therapeutic role in both providing direct treatment services and enhancing web based services. Pragmatic realities may require the softening of the current bias for face to face counselling (Brenes et al. 2011), adjusting our sights in our efforts to be responsive to client needs. Phone counselling appears to have a legitimate place in the continuum of care.

Another option to improving the accessibility of treatment services is web-based counselling. Research into the strengths and appeal of on-line web support is relevant, especially for women. Helplines typically accessed through toll free phone lines now have a larger presence on the web, with added features of on-line (email) contact and real time chat options (instant messaging). In an expansion of services, some web sites offer forums and chat rooms for gambling clients (e.g., Gamcare in the UK, Gambling Help Online service of Australia, and the Ontario Problem Gambling Helpline).

Griffiths (2009) argues that the acceptance of web-based services is imperative in this day and age. Online therapy, he notes, needs to be incorporated within the overall framework of clinical assistance as it may be the only way some people either can or will get help. One benefit of the internet as a medium for help is the *disinhibiting effect* on users and a reduction in efforts to create social desirability (i.e., users do not alter their responses in order to appear more socially desireable; Griffiths 2009). This may lead to increased levels of honesty and, therefore, higher validity in the case of self-disclosure (p. 4). In like vein, Rochlen et al. (2004) write that the on-line context circumvents a person’s overt persona, and clients tend to cut to the chase of core issues (p. 271).

As with phone intervention, the internet is perceived by many users to be anonymous and non-threatening. It may, as a consequence, provide access to ‘socially unskilled’ individuals who may not have sought help if it were not for the online nature of the self-help group (Griffiths 2009, p. 4).

The internet option appears to be especially appealing to women. Data collected from 1200 online counselling sessions by Tyseen (2007) in Australia, reveals that the majority (68 %) involved women. Nearly 70 % of the sessions occurred outside of ‘traditional’ business hours. The most common reasons for online access included privacy (67 %), convenience (56 %) and preferred medium (49 %). Griffiths (2009) similarly observes that the on-line *GamAid* service in the UK may be appealing more to women than comparable services.

As with phone interactions, there is evidence that internet interventions for mental health and addiction can be as effective as face to face therapies (Cunningham et al. 2014. Griffiths (2009) writes that “Online relationships can be as real and intense as those in the face-to-face world (p.2). Gainsbury and Blaszczynski (2011) promote online self-help programs as an alternative means to access treatment for gamblers reluctant to pursue traditional options.

Despite the growing interest in the field, research and development of services is needed: “To date, the limited studies carried out (mostly with very small sample sizes) have focused on client and provider satisfaction with the technology rather than the effectiveness of the technology in delivering services” (Griffiths 2009, p.12). In addition, according to Cooper and Doucet (2002) most of material available to problem gamblers seeking treatment services through the internet is merely information dissemination as opposed to being clinically relevant.

Cooper (2000, 2001a, b) notes the value of informal peer support via web chat forums. Many gamblers, unable to confide in significant others for fear of consequences, feel isolated, shamed and stigmatized. The anonymity of the web offers a mantle of safety. Even simply “lurking” (reading the comments of others without contributing) may assist gamblers move through the Stages of Change described by Prochaska et al. (1994). Many eventually begin to contribute, and some find their way into treatment or Gamblers Anonymous. Cooper (2000) and Wood (2008) note the high proportion of women who participate and their tendency to self-disclose more freely than men. Significantly more females than males suggested that the forums helped them to cope better with their gambling problems. Consistent with the identified appeal to women of on-line services, Albanese et al. (2011) recommended online services be developed as a means of reaching Ontario women who may be geographically isolated or facing significant barriers to treatment and support. This pilot aimed to realize that recommendation.

## Meeting the Needs of Ontario Women Gamblers

As noted, Suurvali et al. (2008) underscore the value of providing a range of treatment options, describing “a growing body of literature supporting the effectiveness of brief interventions for gambling problems offered by telephone or the internet or in the form of self-help workbooks” (p. 1345). With this statement they capture the essence of the pilot study reported in this paper.

This project is part two of a two-part pilot. The first part (Boughton et al. 2016 evaluated feedback for a self-help workbook that was mailed to participants. The results indicated that the workbook was well received by the clients and helped with making changes to gambling, but quantitative measures of its impact failed to reach significance. A drawback to individual phone counselling and self-help workbooks is that they do not provide women the invaluable network of support offered by a group experience. Sharing with peers has transformative power—the normalizing process serves to reduce shame and isolation and assists in the development of mindful self-compassion. In the second part of the pilot, this current study, group counselling, using telephone and web-based services facilitated by a clinician, offered clients clinical support and education. The workbook was used to augment the treatment. Our study aimed to pilot test an online treatment intervention heretofore not offered to women who gamble.

The method chosen was to run the study as a pragmatic clinical trial (see Tse et al. 2013). Pragmatic clinical trials investigate how effective an intervention is in everyday practice (MacPherson 2004). We had three hypotheses: 1) that women in the program would make positive progress on their stated gambling goals when comparing their pre-program scores to their post-program scores; 2) that participants would report positive experiences of personal growth and learning in their quantitative feedback to the program; and 3) participants would report positive experience of personal growth and learning in their qualitative feedback to the program.

## Methods

This study explored the viability and effectiveness of a clinician-facilitated group using teleconferencing and visual access through Adobe Connect. The Research Ethics Board of the Centre for Addiction and Mental Health (CAMH) approved the research as protocol # 153/2012.

### Participants

A total of twenty-five women participated in five webinar groups. Recruitment ads were placed in small local newspapers around Ontario, as well as on various web sites related to information on problem gambling, including the Ontario Gambling Helpline. Participants were paid a total amount of $165.

Participants were screened by phone-intake interview. Inclusion criterion included women identifying gambling concerns but not currently in formal gambling-specific treatment; proficiency in English (reading, writing and verbal); ability to fully commit to time demands of weekly participation; interest in the pilot treatment; access to internet and telephone including use of e-mail to send materials.

Exclusion criteria included mental health concerns of sufficient severity to compromise weekly involvement in group (i.e., active psychosis, unstable mood disorders or extreme social anxiety); cognitive challenges in reading materials or completing written assignments; suicidal behaviours; drug/alcohol abuse that would interfere with participation. Women not eligible were offered information on appropriate services when possible.

The group webinar consisted of 12 webinar sessions over a three-month period. Five group webinars were conducted between late November of 2013 and November of 2014. Twenty six (*n* = 26) potential participants were assigned to a group. Eight (*n* = 8) never attended any session bringing the actual webinar sample to 18. Eleven women (*n* = 11) completed, most of the 12 webinar sessions and the final feedback forms, for an attrition rate of 7/18 or 38 % of those who started the group. The known reasons included scheduling difficulties, work conflicts, health problems and computer problems.

Most women (85 %) were of white European descent. The average age was 56 with a range of 28 to 70 years (*SD*: 9.7). Less than half (42 %) were married or in common-law relationships. Those in a current relationship (*n* = 11) reported durations ranging from 4 years to 37 years in length, with an average of 21 years. On a scale of 1–10 women ranked how satisfied they are with their current situation or relationship. Responses ranged from 4 to 9 with a mean score of 6.5. A minority (26 %) reported having from 1 to 2 children living in the household. The largest percentage (46 %) had completed some university education or a professional degree, while 20 % had a high school education or less and 16 % a community college or technical school. The majority worked full or part time (56 %) or were looking for work (8 %) while 8 % were on disability and 27 % were retired.

### Pre-Study Instruments

Initial pre-screen materials, formal consent to treatment form, and limits to confidentiality were mailed to women interested and eligible to engage in the study. Responses were signed and returned as required by Ethics protocol. Correspondence throughout the remainder of the study was via email. Both quantitative and qualitative data were gathered with the pre-study package. Demographic information was collected, including family history, personal history of drug or alcohol use, struggles with other behaviours (i.e., eating, shopping), mental health status, trauma history and gambling behaviours (triggers, patterns, type). The package included some standardized gambling, mental health, and quality of life and well-being measures. Gambling difficulty was assessed using the Diagnostic and Statistical Manual of disordered gambling (DSM IV) (Slutske et al. 2011) and the Canadian Problem Gambling Index (CPGI) (Ferris and Wynne 2001). As women’s gambling is often associated with efforts to escape emotional stress and isolation, and self-esteem can be an issue, the women completed screens targeting these areas.

### Post Treatment Measures

The DASS-21 (Lovibond and Lovibond 1995) screens for depression, anxiety and stress. The Perceived Stress Scale (PSS) (Cohen et al. 1983) is designed to measure the degree to which situations in one’s life are appraised as stressful. In order to conduct pre-test post-test evaluations the PSS and the DASS21 were administered before the first session and after the last session.

Following each session, participants were asked to provide feedback ratings on different aspects on the module, report on any gambling activity, and indicate their gambling treatment goals.

After the final session, additional feedback forms asked for comments on their experiences and overall impressions of the Tutorial Workbook; and to note whether participation in the study impacted beliefs and attitudes about gambling and gambling behaviours, and if and how the workbook was helpful. The open ended feedback was the basis for the qualitative analysis presented below.

### The Tutorial Workbook Modules (TW)

The treatment group utilized the Tutorial Workbook (Boughton 2015; Boughton et al. 2016) for the educational and practice component of the webinars. The weekly modules, forms for self-monitoring and feedback evaluations were offered in PowerPoint format. The participants were asked to review the materials, complete the module worksheets, and email the completed feedback to the facilitator prior to the next scheduled webinar meeting. The feedback pages asked participants to both report on their goal progress and provide input on the materials of each module, as well as a rating scale to evaluate the module.

### The Webinar

Group meeting days and hours were negotiated with potential group members to optimize access. Five groups engaged at different times and on different days of the week. Once enough women were potentially gathered to form a group (8 members) participants were emailed information about the dates and the process of logging in to the Webinar. A couple of days prior to the scheduled group the women were sent an email invitation through Adobe Connect which provided them with the phone number and the web link to access the webinar. Confidentially of email contact information of the various recipients was ensured by sending it in the form of a blind copy.

The webinar combined structured and semi-structured elements. The facilitator began with a brief Mindfulness meditation, followed by administrative tasks such as reminders to send in feedback forms. For about the next hour, members reported on progress towards their gambling goals, including triggers or urges over the week, and described their overall well-being with discussion and input from group members and the facilitator. To maintain confidentiality within the webinar, participants were asked to use only first names, and to refrain from sharing contact information or divulging private information about other group members. The facilitator then reviewed the module topic for the week. A modified version highlighting some of the key ideas, simulating a flipchart, was uploaded to the Adobe site, allowing materials to be visible during the discussion through the web link. In addition to the conference call, group members had the option to type in comments or raise their hands. The facilitator was able to review the materials, elicit thoughts, explore worksheet responses, and answer any questions or concerns generated by the materials. At the end of the session, participants were asked how they were feeling and what learning or awareness they were taking away from the group meeting. Finally, they were asked via email to complete an on-line SurveyMonkey questionnaire which specifically addressed their experience of the webinar process and content. Immediately following the 1½ to 2 h. hour webinar, group members were emailed the TW Module to be discussed the following week.

## Data Analysis

### Quantitative Analysis

The demographic, history and background information is reported in terms of frequencies. For the PSS (The Perceived Stress Scale, Cohen et al. 1983) and DASS-21 (Depression, Anxiety and Stress Scale, Lovibond and Lovibond 1995) the data is analysed using repeated measures t-tests.

### Qualitative Analysis

A qualitative analysis was utilized to provide an understanding of women’s perspective of the workbook contents and webinars. NVIVO10 software (QSR International 2012) was used to generate an inductive grounded theory approach. This framework was chosen to allow the emergence of concepts/categories from the data itself. The narratives of women were read and coded with no preconceptions and upon reading participant responses. Codes, categories, themes, and outlier data were identified in this process (Creswell and Maietta 2002). The data from the workbook and webinar were analyzed by a co-investigator (FJ) who was not involved in data collection.

Braun and Clarke (2012) outline a series of phases through which researchers conduct thematic analysis. The initial coding phase involved coding each question of the survey across all the modules. This resulted in the emergence of 530 codes from the collected data. Various themes were revealed through analysis of these codes. A full coding pass allowed for the emergence of themes.

Perspectives related to gambling behaviours and triggers, emotions, moods and relationship issues were identified and coded into key issues, themes and sub-themes. On the basis of this analysis, a thematic framework was developed. The raw data was again examined using Nvivo10 software to ensure the robustness of the analytical process and to confirm that all data were indeed reflected in the coding. A refined coding scheme was developed and used to explore the data, increase understanding of the data, test-coding rigour, and to develop and understand thematic relationships of participant responses.

Following the extensive coding process, four primary dimensions emerged: (1) dealing with gambling, (2) improved coping, (3) positive psychological impact, (4) decreased isolation. These dimensions included themes and sub-themes. Each participant response per module was coded to develop a file of references made to the theme and sub-themes of gambling, psychological impact, coping and decreased isolation.

## Results

### Family and Personal History

#### Abuse and Trauma Histories

The participants (*n* = 25) shared information about their childhood and adult experience of abuse and trauma. They revealed high levels of emotional abuse both as children (59 %) and adults (70 %). Sexual abuse was also reported as adults (31 %), and as children (19 %). Physical abuse is reported at lower levels as children (19 %) than as adults (27 %). Experiences of loss were high (as children 35 %, as adults 58 %) as was trauma (as children 31 %, as adults 31 %). These rates of abuse and trauma are at rates higher than in the general population.

#### Abuse in Current and Past Relationships

As seen in Table 1 the women in current relationships report spousal struggles with issues such as drugs or alcohol (18 %), and mental health (9 %), but none with gambling. The predominant form of abuse reported is emotional (29 %). This stands in stark contrast to the reports about spouses from past relationships (*n* = 8) where emotional abuse is reported at 63 %. On average these women had been separated for 14 years but it ranged from 3 to 32 years. They report higher levels of abuse, anger, criminal convictions, and drug and alcohol spouse problems in past relationships.

**Table 1:** Relationship problems (*n* = 25)

Relationship	Current Relationship %	Past Relationship %
Problem of Spouse/Partner	(*n* = 11)	(*n* = 17)
Drugs or alcohol	18	18
Mental Health	9	18
Gambling	0	9
Anger	9	36
Criminal Convictions	0	18
Abuse in Relationship	(*n* = 7)	(*n* = 8)
Physical	0	25
Sexual	0	25
Emotional	29	63

#### Mental Health Concerns

A large number of participants reported having had professional treatment for mental health issues: depression (42 %), anxiety (31 %), panic (12 %), manic-depression (8 %), or anger (12 %). More than a third (36 %) had been prescribed medications for emotional issues and a number were currently taking medications (12 %). Many acknowledged serious thoughts of suicide (23 %) but none acknowledged attempts (0 %). A few (12 %) had been hospitalized for mental health issues.

#### Co-Occurring or Past Problematic Behaviours

A large proportion of the women also report dealing with other problem behaviours, past or current (see Table 2). This may be a result of redirecting the money and focus to gambling.

**Table 2:** History of substance use and behavioral problems (*n* = 17)

	Past Problem (%)	Current Issue (%)
Alcohol	23	4
Prescription drugs	4	0
Non-prescription drugs	12	0
Smoking	31	0
Shoplifting	12	0
Compulsive shopping	31	8
Sexual behaviour	23	4
Eating disturbances		
Binge eating	35	19
Starving self	8	8
Aggressive behaviour	4	0
Criminal behaviour	8	0

## Gambling Issues

### Types of Gambling

Slot machines (73 %), lottery games and scratch tickets (31 %) were the games played by the largest proportion of the women. On average they played 3 different games each month, with a range from 1 to 6 games. There was considerable variety in the games, likely related to access as most of the women involved in the webinars were living in the USA, including one woman living in Las Vegas.

The women spent, on average, 74.9 % (*SD*: 76) of their net personal income on gambling every month.

Fifty eight percent (58 %) accumulated debts related to the gambling through credit card (58 %), bank loans (50 %), unpaid bills (50 %) and borrowing from family (39 %). A number also pawned or sold personal property (31 %), declared bankruptcy (11 %) and cashed in RRSP (12 %).

### Drawbacks to Gambling

Participants reported drawbacks to their gambling from a list of 60 items. Table 3 contains the 25 items that topped the list as drawbacks as often or always an issue. The list is dominated by financial concerns and emotional distress.

**Table 3:** Drawbacks to gambling (*n* = 24)

	%
Anger at myself or others	96
Stress over money loss	95
Guilt	92
Secrecy about the time or money spent	92
Losing money I can’t afford	88
Worry about my financial future	87
Depression as a result of gambling	79
Feeling out of control	79
Worry	75
Fear/anxiety related to gambling	75
Difficulty sleeping	71
Breaking promises to self or others	67
Debts as a result of gambling	67
Loss of self-esteem	67
Gambled whole check	58
Taking money from other things	54
Spending savings or inheritance	54
Not taking care of myself	54
Interest charges on credit cards	50
Losing the trust and respect of others	50
Lying or manipulating	50
Time away from friends or family	46
Borrowing money	38
Tensions or arguments with others	35
Confrontations about money spent on gambling	33

### Problem Gambling Indicators

#### CPGI

The Canadian Problem Gambling Index (CPGI, Ferris and Wynne 2001) was used to screen participants for levels of problem gambling. The level of problem gambling was high, with 91.7 % (*n* = 22) scoring as problem gamblers and the remainder (8.3, *n* = 2) having moderate problems (8.3 %, *n* = 2). The predominant 1st or 2nd game of choice was slot machines and 94 %, (*n* = 17) of these participants scored as problem gamblers, the other participant (*n* = 1) scoring as having moderate issues. All the women playing video poker (*n* = 3) scored as problem gamblers, as was the case with the roulette (*n* = 1), keno (*n* = 1) and horse racing (*n* = 1). Three women gambled at card games, two at a problem level and one at a moderate level of problems.

#### DSM IV

The DSM-IV diagnostic criteria for pathological was also included among the screens. However, given the recent publication of DSM 5 we used the DSM-5 scoring, rather than the DSM-IV scoring using the scoring suggestions of Turner et al. (2016) The women ranged in scores on the DSM 5 from 0 to 9 with an average of 6.0 (*SD* = 2.7). Most (83 %) of the women scored as disordered gamblers and another 8.3 % scored in the subclinical range.

### Pre and Post Study Evaluation Data

Four scales were used to evaluate the impact of the program on the participants: the Perceived Stress Scale (PSS) and the depression, anxiety, and stress subscales of the DASS-21. The means and standard deviations for these variables are given in Table 4.

**Table 4:** Pre (T1) and post treatment (T2) scores for the PSS and DASS including subscale scores

Variable	*N*	Mean	*SD*	*T*	*p*	*D*
PSS Total T1	11	19.9	5.7	2.85	0.02	0.86
PSS Total T2	11	14.5	7.1			
DASS Depression T1	11	5.7	5.8	2.23	0.05	0.67
DASS Depression T2	11	3.5	6.2			
DASS Anxiety T1	11	1.8	2.3	−1	0.34	−0.3
DASS Anxiety T2	11	2.4	3.3			
DASS Stress T1	11	4.2	3.6	−1.29	0.22	−0.39
DASS Stress T2	11	5.7	4.9			
DASS Total	11	11.7	8.8	0.09	0.93	0.03
DASS Total	11	11.5	13.2			

*t* the *t*-test value, *p* the probability estimate for the *t*-test, *d* = the estimated effect size

#### PSS

The Perceived Stress Scale (Cohen et al. 1983) was used to measure stress. Scores around 13 are considered average. Scores of 20 or higher are considered high stress. Eleven (11) women completed both pre and post screens. In the pre-study measures the average score was 19.9 (*SD* = 5.7). The post study scores show an average of 14.5 (*SD* = 7.1; *n* = 11). The SPSS results of a *Paired Samples Test* comparing the pre and post scores indicates that the mean initial score was significantly lower, *t* = 2.85, *p* = .02. The effect size was *d* = .86.

#### DASS-21

The DASS-21 (Lovibond and Lovibond 1995) was completed as part of the pre and post study instrumentation. Analyses were conducted on the raw scores for the subscales of depression, anxiety and stress. The results indicate a decrease in depression from pretest to post-test that just barely reached significant, *t* = 2.23, *p* = 05. The effect size was *d* = .67. The results for the DASS anxiety, DASS stress scores, and the DASS full scale, were not significantly different.

### Feedback on the Tutorial Workbook (TW)

The 12 modules of the TW address both gambling specific issues and those commonly underlying problematic gambling for women (Boughton 2015; Boughton et al. 2016). The first few explore whether gambling is a problem, establish gambling-related goals, and address relapse prevention strategies. They include topics such as supporting the change process and dealing with urges to gamble and/or lapses. The remaining modules shift to a focus on issues often lurking below the surface of the gambling urges. The modules also include exploration of how our thoughts impact our feelings, mindfulness, stress management, relationships, and regulating emotions.

### Quantitative Feedback on the TW

Each Module included an identical feedback form, using a Likert scale to gather the women’s thoughts: 1 = Strongly Disagree, 2 = Disagree, 3 = Neutral, 4 = Agree, 5 = Strongly Agree. Two of the seven items were framed in negative terms. As shown in Table 5, all of the positively keyed feedback items were endorsed by a majority of the participants, whereas none of the negatively keyed feedback items were endorsed by more than 15 %. For the sake of simplicity, we have only reported the number of women’s who agreed or strongly agreed with each of the evaluation statements.

**Table 5:** Number of people who agree or strong agree which each of the feedback items

	1	2	3	4	5	6	7	8	9	10	11	12
	17	16	14	11	9	11	12	11	8	10	8	9
I found this module easy to understand clear	94	88	93	100	100	100	67	82	88	90	100	100
This module had too much information	0	6	21	0	0	27	8	9	38	20	13	0
I liked the graphics and layout.	59	88	86	82	78	82	83	92	75	70	88	56
I found this information relevant to me	84	100	57	100	100	100	92	100	75	90	100	89
Overall I found this module helpful	88	100	71	100	100	82	92	91	75	90	100	100
I would recommend this module to a female friend in my situation	88	100	72	100	100	91	75	91	100	90	100	89
I was very disappointed with this module	0	0	7	0	0	0	0	0	0	10	0	0

### Changes in Gambling as a Result of Involvement

The feedback forms gathered information on the women’s initial gambling goals and their progress on those goals. This included monitoring changes in urges, and in types of gambling, frequency and amounts gambled over the course of the study.

The majority (67 %) of the women wanted to stop all gambling while others wanted to limit the frequency (11 %) or stop certain games (11 %). Most (89 %) reported a decrease in frequency of play. Forty-four percent (44 %) also reported a decrease in the amount of time they spend when they gamble, while 33 % indicated no change. A fifth (22 %) reported that they spend less money when they play, while 33 % report no change in how much they risk. A quarter (25 %) of the women reported that they have stopped certain forms of gambling as a result of participation in the study, while 38 % report no change in the types of games. Over half (56 %) reported a considerable reduction in urges; 33 % of the women report that they still have urges but are better able not to act on them. Eleven percent (11 %, *n* = 1) indicated that the thoughts/urges are as strong and intense as before. Reflecting on the change process, women were provided with a list of both positive and negative options: 33 % (33 %) indicated that they have been successful in making the changes they wanted. A further 56 % indicated, “Overall, I feel I’m making positive progress”.

### Benefits of the Treatment

Participants were asked to reflect on the overall impact or benefits of being involved in the pilot. They were given a series of statements focused on self-esteem, moods, isolation and understanding and asked to complete a Likert scale: 1 = Strongly Disagree, 2 = Disagree, 3 = Neutral, 4 = Agree, 5 = Strongly Agree. The results, indicated in Table 6, were positive in that the majority agreed or strongly agreed with each of the statements. All (100 %) of the women reflected both a better understanding of triggers/issues and gaining more tools of awareness and choices as a result of the learning. Ninety percent (90 %) reported that it had helped with moods and anxiety levels and that they felt more hopeful about the future. Over 80 % reported improved relationships (82 %) and feeling better about themselves (80 %). Seventy-three percent (73 %) reported feeling less isolated and alone with the issue.

**Table 6:** Benefits of the treatment (*n* = 11)

Benefits of the Treatment	Agree	Strongly Agree
I think I have a better understanding of my gambling triggers and issues	54	46
I have more tools of awareness and choices as a result of what I have learned	18	82
I am feeling better about myself	46	34
I think it has helped my moods and anxiety levels	50	40
I feel less isolated and alone with this issue	27	46
I have been able to improve my relationships with what I have learned and changed	73	9
I am more hopeful about the future	36	55

### Survey Monkey Webinar Feedback

Following completion of each weekly group, webinar participants were also asked to complete an on-line feedback form (Survey Monkey) that collected a mix of quantitative and qualitative data. Space was also provided to articulate thoughts and responses to the session content and process. As shown in Table 7 a large majority of the participants agreed that each of the webinars were helpful. Two notable exceptions, however, are for the length of the webinar for Modules 9 and 10. It is not known if they found the webinar too short or too long but given the amount of materials in these latter modules, and comments to the facilitator during the webinar discussion, it is presumed that they wanted more time for discussion (i.e., additional sessions on the topic).

**Table 7:** Feedback from survey monkey for the weekly webinars based on survey monkey data

Module	*N*	Mindfulness Helpful	Length of Webinar good for me	Webinar Content Beneficial
1	16	100 %	94 %	100 %
2	16	94 %	88 %	100 %
3	11	100 %	100 %	100 %
4	5	80 %	100 %	100 %
5	11	100 %	100 %	100 %
6	14	93 %	93 %	100 %
7	12	100 %	83 %	93 %
8	12	100 %	100 %	100 %
9	11	100 %	55 %	100 %
10	13	100 %	37 %	100 %
11	10	90 %	100 %	100 %
12	11	100 %	100 %	100 %

In addition, the participants provided written feedback comments about the webinars. The following comments highlight participant satisfaction with the TW modules:

“I was prone to self-criticism when I had lapses/slips. With more self-knowledge I tend to examine how I am feeling or when I am feeling uncomfortable with myself. I can now generally work through the feelings and avoid lapses/slips before they take off past a manageable point” (Module 5).

“I thought it was good to learn what my thinking patterns were and the importance of recognizing self-talk. I liked the idea of changing the way I talk to myself, look out for all or nothing thinking and practicing mindfulness to increase compassion toward myself” (Module 8).

“Each week, I feel more and more empowered by the information we receive and review. The information regarding stress as well as the other modules helps me see that it is a series of small choices, ways of thinking, responding, etc. that leads to BIG changes. The modules help me to see the possibilities for growth in many aspects of my life. I am very grateful that I got to be part of such a wonderful experience” (Module 11).

“As we work toward changing habits and behaviours, 12 weeks is not enough. Our gambling addiction took much longer to create. We cannot accomplish full change in 12 weeks” (Module 12).

Some women in sharing their feedback to the webinars expressed that they found the information to be valuable but too much for 12 sessions. They expressed that a longer webinar series with subsequent break down of content would make the modules less of an information overload. In the final question of the module, asking how the module could be improved, one participant stated: “I think the members should have the option to contact each other for support” (Module 5). This statement highlights the importance of on-going peer support to the participants.

### Qualitative Data

A purposive data analysis technique with the coding scheme described earlier was used to provide a qualitative analysis: dealing with gambling, improved coping, positive psychological impact, and decreased isolation.

#### Dealing with Gambling

The women reported that they were learning new and valuable ways to deal with their gambling. Many acknowledged the influence that it had in their lives and determination to change. “I know that I have a serious issue with gambling and do want to change this. I am incorporating websites, mindfulness, stress and relaxation and protective methods, not carrying money etc. to stay safe and away from gambling. I have developed an ongoing treatment plan for myself and recognize my abilities as well as the liabilities I still need to address” (Participant #731).

Many had been involved in other forms of gambling treatment, but had not been successful because of the insistence of abstinence. In contrast they found the exposure to a harm reduction philosophy in this treatment empowering.

#### Improved Coping

As women were beginning to identify and become more aware of triggers and urges to gamble, learning to take steps to proactively change their gambling related behaviours, many spoke about the development of new coping strategies: “At this point in my recovery, I find reviewing strategies and tools, especially on avoiding relapses very beneficial. It helps keep me vigilant and aware that I shouldn’t take recovery for granted – it is something that has to be continually worked at” (Participant #737).

Thus many women expressed that through participation in the webinar, they were learning new tools and strategies that they could utilize. Coping and the sub-themes of self-compassion and emotional awareness were frequently discussed by women in the groups. For many, a change in gambling behaviours was linked to awareness of emotions and stress related triggers:

“I am trying to accept that not everything is in my control…. When I am upset, I go to another room to pause and think about why I am angry and how relevant my anger is to the situation. This pause helped me to think logically, calm me down and change my attitude in the process (Participant #716).”

Women shared examples of situations in their lives when they had implemented strategies learnt through the tutorial workbook and webinars in challenging times in their lives. They reported being able to generalize the coping strategies to other areas of their lives. Participants expressed that they were attempting to regularly incorporate meditation into their lives and this was particularly pertinent when experiencing low mood. The regular practice of mindfulness and the stress reduction strategies were reported as especially beneficial by some of the women. They wrote of experiencing more positive benefits than they had gleaned from other forms of treatment.

#### Positive Psychological Impact

The psychological impact of the webinars was related to sub-themes of self-esteem, hope regarding the future, new coping strategies, social support from the group and program perseverance. Greater confidence and happiness was linked to having more hope about the future and feeling that life in the absence of gambling was attainable. As women discussed greater hope, they were taking steps for self-care, having greater compassion toward themselves, became more aware of emotions and behaviours and able to remember to implement the strategies learnt during the course of the webinars:

“I found it helpful to review how to be kind to myself. It’s a good reminder that I don’t do it often enough. I am now taking time to rest, taking hot baths to relax. I said NO to doing something I didn’t want to do. I took better care of myself. I am giving myself grace” (Participant #702).

At program completion, women reflected on the process of the webinar series and their involvement in the study. Some shared that program participation had been challenging at times and, despite considering quitting the program, they felt proud of their commitment to the program as they were now beginning to experience the benefits:

“There were times when I was going through the program that I felt hopeless but sticking with it and gaining some time away from gambling has really made me feel hopeful again. It was around week 9 or 10 I think that I felt a shift and started to focus more on what I want in my life and actually started to believe that I can have it. But I can only get there if I quit gambling” (Participant #733).

Program commitment was also linked with feeling greater hope and feelings of resilience. For the first time, many were considering life in the absence of gambling.

#### Decreased Isolation

Positive changes were strongly linked with feeling supported by other women in the group process. A feeling of connection with other women was cited as extremely important. For many women in the group, this was a new experience. They felt less alone in their addiction and in their feelings. The group offered a supportive environment to both learn new gambling related information and coping strategies and also provide a safe space to discuss other pertinent issues connected to gambling. The group social support and sharing was crucial to enhancing a sense of wellness.

“I haven’t felt isolated or alone for quite a while. Listening to other participant’s stories and struggles reminds me I am not alone. I don’t have to die of shame off in a corner. During the group, I was comfortable to say what was true for me and hopefully I can stay connected with the group members” (Participant #709).

A changing relationship with self was associated with a changing relationship with others and less loneliness. Greater knowledge was related to making different choices associated with gambling.

Overall, women participating in the webinar group shared that they were learning new tools for their gambling, accepting the influence of gambling in their lives and the implementing new strategies to deal with it.

In addition to the benefits in the four domains described earlier, the webinar group interactions were perceived as highly beneficial in consolidating the learning offered by the workbook modules. The women were able to share their experiential wisdom, teaching, inspiring and supporting each other. They felt safe. They felt heard and understood. They felt validated and empowered by the joint experience. They felt less isolated in their struggle, less alone. They gleaned new awareness regarding their gambling and triggers, sharing their successes in applying the coping strategies and making changes. For many, the treatment was the first time learning of a harm reduction perspective. The women felt more hope and improved self-esteem as they made healthier choices and began to practice more self-compassion. As was evident in their narratives, the webinar group was thought to be a critical component of the treatment program.

## Discussion

This project was the realization of a need noted by Boughton and Brewster (2002) for treatment options designed for and geared towards women who have difficulty accessing on-site treatment services due to a number of possible barriers.

The aim was to test the effectiveness of a treatment protocol that combined teleconferencing with a webinar in a group format. The treatment design built upon current gambling literature that validates the treatment effectiveness of self-help material, phone counselling services, and internet-based treatment. It further evolved from clinical research describing barriers to treatment for women, the distinct treatment needs and issues of women gamblers, and the value of group counselling for women. The research project consisted of two independent pragmatic pilot studies. The first, reported by Boughton et al. (2016), explored the use of a tutorial workbook (TW) that was developed at CAMH and drawn from best practice resources in mental health and gambling treatment. It was mailed to Ontario women in rural populations recruited through small town newspapers.

The second, reported in this article, explored the effectiveness of a clinician-facilitated group treatment protocol using teleconferencing and a webinar in combination with the tutorial workbook. It was offered to women gamblers in North American communities who responded to internet recruitment. Both interventions are new innovations in the area of problem gambling treatment.

Research participants effectively served as pseudo focus groups to get experiential and practical feedback from women gamblers on both the content and structure of the Tutorial Workbook itself, and to gather input on the effectiveness of the webinar in providing additional support.

In terms of clinical outcomes, the researchers anticipated positive progress on the stated gambling goals of participants, positive personal growth and learning, improvement in terms of the perceived stress (PSS) and depression, anxiety and stress (DASS-21). It was also anticipated that the webinar group experience would add an additional opportunity for learning and growth beyond that offered by the actual contents of the workbook. As reported in the foregoing, there is strong evidence that the webinar did meet these expectations; participants consistently reported that both the group experience and the workbook materials were valuable, and this cuts across all of the modules and webinar sessions.

Of the four scales that were used evaluate the impact of the group comparing pre and post treatment scores, two showed some evidence of an impact: the PSS stress score and the DASS-21 depression score. These findings are weak and need to be replicated. However, they are promising.

The qualitative data also contributed to understanding the impact of the webinar and workbook combination. Women conceived that participation was related to changes that extended across four primary dimensions: (1) dealing with gambling, (2) improved coping (3) positive psychological impact, (4) decreased isolation.

The webinar offered an opportunity to develop new awareness and coping strategies regarding gambling behaviours, triggers and urges. Participants found Mindfulness training particularly valuable, sharing that the meditation practices helped to calm them, deal with triggers, and learn to live in the present moment even when urges to gamble arose. The women also spoke of the empowerment offered by a harm reduction perspective, reporting increased self-esteem, greater hope, less emotion dysregulation, and reduced isolation. They reported new gambling-free behaviours and a greater happiness of financial management.

In essence, the webinar was value added to the workbook materials. For many, this was the first opportunity to learn that other women were experiencing gambling-related issues and to talk about it openly. The women shared that the webinar component was critical to fully engaging in the content of the TW. Having the shared space with other women and a skilled facilitator offered the opportunity to go into greater depth with the TW material. In this context, women were provided additional support and could ask questions, grapple with the material and fully think about how to implement the material in their lives. The interactive experience of the group contributed powerfully to learning self-compassion, self-care, feeling less isolated with the gambling issue and less alone. It reduced shame and guilt and created space for compassion and understanding. Related to this positive experience, some women recommended more time for the webinar series and the possibility of an extended group. Some expressed a desire to stay connected with group members. One participant shared that she was grieving the end of the group as she had benefitted so much from the group interaction. These shared communications demonstrate the impact of the webinars in the lives of the women.

## Limitations

There are several limitations to this treatment project. The sample size was small (*N* = 18) with a high attrition rate (39 %). As not all participants completed all of the questionnaires, so there were missing values in the data set. Specific to screening, there was no formal diagnostic screening of mental health or problem gambling issues. The intake screening was carried out over the phone and information was provided by self-report. These factors add additional uncontrolled variance to the study’s results.

The scope of the webinar study expanded to include women anywhere in North America, possibly by using the internet for the webinar, telephone conferencing to provide audio group link, and a visual connection through a web link. The cultural difference between Canada and the US may limit the ability to generalize the results to Ontario populations of women gamblers. Although pragmatic research is thought to monitor effectiveness, the efficacy of the results cannot be ascertained as this was not a controlled study using random assignment to a control or treatment group. Thus, despite the overall preliminary positive findings of this pilot treatment study, more research is needed to fully evaluate the impact of the workbook or webinars.

## Further Considerations, Next Steps and Conclusions

The results of this small exploratory study are promising. Based on the feedback, the contents of the workbook might be shorter and/or the duration of the webinars longer as twelve sessions was insufficient to both provide supportive discussion of issues and cover the salient points in the module under discussion. The high attrition rate was a concern. Although this is not atypical in treatment programs, additional strategies to encourage adherence to treatment, such as using motivational interviewing, commitment strategies, and follow up with clients, might be woven into the treatment protocol. Further research on the efficacy of these interventions would benefit from the addition of a control group. In order to increase sample sizes recruitment efforts will need to be expanded, with more varied Internet social networking and more extensive advertising than was possible in this small study.

The research results encourage the addition to existing services of this alternative mode of treatment. It confirms the value of a self-help workbook for women who gamble, the benefit of a women-only group, the effectiveness of phone counselling and the feasibility of using web-based interventions.

The preliminary data presented here is a positive first step towards filling a treatment gap for female problem gamblers.
